# The Exercise Preconditioning Effect on Cardiac Tissue Injury following Induction of Myocardial Infarction in Male Rats

**DOI:** 10.1155/2023/3631458

**Published:** 2023-07-13

**Authors:** Fatemeh Heiat, Azam Ahmadi, Manzarbanoo Shojaeifard

**Affiliations:** ^1^Department of Physical Education and Sport Sciences, Fasa Branch, Islamic Azad University, Fasa, Iran; ^2^Department of Exercise Physiology, Faculty of Physical Education and Sports Science, Kharazmi University, Mirdamad Ave., Tehran, Iran; ^3^Department of Physiology, Fasa University of Medical Sciences, Fasa, Iran; ^4^Ionizing and Non-Ionizing Radiation Protection Research Center (INRPRC) Shiraz University of Medical Sciences, Shiraz, Iran

## Abstract

**Materials and Methods:**

Twenty-four male rats were divided into 4 groups including MI, Sham, HIIT, and HIIT+MI (*N* = 6). HIIT and HIIT+MI which underwent high-intensity interval training (HIIT) for 4 weeks (5 days a week). The training protocol included 10 intervals of 1-minute running, with 2 minutes rest between each interval. The training intensity was different every week according to the peak treadmill running speed (v peak) percentage of each rat. Isoproterenol injection was used to induce myocardial infarction (MI). Expressions of creatine kinase-MB (CK-MB), PGC-1*α* troponin-I, mitochondrial transcription factor A (TFAM), vascular endothelial growth factor (VEGF), and microRNA 126 (miR-126) genes were measured. The variables were measured using biochemical and RT-PCR methods. The significance level (*P* value≤0.05) was analyzed using ANOVA test.

**Results:**

The results showed that 4 weeks of HIIT training led to a significant increase in PGC-1*α*, TFAm, and VEGF levels in the MI, HIIT, and HIIT+MI groups compared to the sham group (*P* = 0.001). HIIT exercises increased miR-126 in the different groups compared to the sham group; however, it was not significant.

**Conclusion:**

The results obtained showed that HIIT exercise exerts cardio-protective effects to reduce cardiac tissue injury and necrosis against MI. These effects increase mitochondrial biogenesis and angiogenesis by inducing the increased expression of VEGF, TFAM, PGC-1*α*, and miR-126 genes in the heart tissue. Therefore, HIIT training, as a preconditioning program, was able to protect the cardiac tissue against MI.

## 1. Introduction

Myocardial infarction (MI) occurs due to the loss of blood flow as a result of the blockage of the blood vessels feeding the heart muscle [1]. Mitochondria, which is one of the vital intracellular organelles, and its biogenesis process, including the creation of new mitochondria and increased mitochondrial density, are affected by MI. It has been shown that peroxisome proliferator-activated receptor-gamma coactivating factor (PGC-1*α*), as the most important marker of mitochondrial biogenesis, is decreased in the heart following MI [2, 3]. Besides, treatment with angiotensin II receptor blockers (ARB) and PPAR agonists in PGC-1*α* deficient rats improved both ventricular function and PGC-1*α* levels and reduced the damage caused by reperfusion ischemia [4, 5]. Therefore, it seems that one of the protective pathways of the heart against ischemia is the protection of mitochondrial function. In this regard, improving the process of mitochondrial biogenesis can minimize the complications caused by heart infarction [6]. On the other hand, epidemiological studies have shown a strong relationship between people's physical activity and the survival of patients from MI [7]. Physical activity has been shown to not only reduce cardiovascular risk factors but also promote cardiac protection against ischemia-reperfusion and MI [7]. Today, high-intensity interval training (HIIT) is considered one of the useful training methods among sports activities in improving aerobic capacity and cardiac protection [8, 9].

In this context, the results of related research have shown that (PGC-1*α*) is upregulated by HIIT exercises [10–12]. Researchers attribute the improvement in aerobic capacity to mitochondrial biogenesis. It seems that PGC-1*α* is directly involved in the production of nuclear respiratory factor 1,2 (NRF-1,2) and mitochondrial transcription factor A (TFAM) [13]. This gene (TFAM) is also a mitochondrial transcription factor that is a key activator in mitochondrial transcription [14–16]. Also, studies show that PGC-1*α* plays an important role in stimulating angiogenesis caused by exercise through increasing the expression of vascular endothelial growth factor (VEGF) through the activation of the ERR*α* pathway [17–19].

Angiogenesis means increasing the density of cardiac and skeletal muscle capillaries [20] in response to such stimuli as hypoxia [21], hemodynamic forces (shear stress, tissue mechanical stretching), and metabolic factors (including growth factors) [22, 23]. VEGF increases the immigration and proliferation of endothelial cells. The formation of a vascular network, as the strongest and most important factor affecting angiogenesis, is necessary for the differentiation of endothelial cells and the germination of new capillaries from previous vessels [24, 25].

Also, recent studies have shown the important role of microRNAs in the response of the cardiovascular system to injury, inflammation, and stress [26]. miR-126 is known as an endothelial-specific miRNA. It plays the most important role in controlling angiogenesis and vascular integrity [27]. Da Silva et al. studied the role of swimming aerobic exercise in the expression of miR-126, showing that aerobic exercise can increase the expression of miR-126. This increase can be related to cardiac angiogenesis caused by exercise activity by directly regulating the VEGF pathway and indirectly regulating its targets such as MAPK and PI3K/Akt/eNOS [27]. Physical activity interventions are recognized as a safe, low-cost, and important strategy for the prevention and treatment of cardiovascular diseases. Recent data suggest that HIIT has greater benefits than endurance exercise in improving cardiac performance [28]. Regarding the effect of physical activity on mitochondrial function and its positive effects on myocardial mitochondrial biogenesis and angiogenesis caused by it, this question arises whether HIIT exercise (intermittent and intense) for 4 weeks reduces the cardiac tissue injury by increasing myocardial mitochondrial biogenesis and by activation of angiogenesis signaling pathways due to the changes in mitochondrial dynamics (expression of PGC-1*α*, TFAM, VEGF, and miR-126 genes).

## 2. Materials and Methods

### 2.1. Animals

Twenty-four male Wistar rats aged 18-20 weeks (weight: 272.73 ± 23.74 g) were obtained from the Pasteur Institute of Iran. They were kept in polycarbonate boxes (three rats in each cage) in the standard environment (temperature of 22 ± 1/4°C, humidity of 50% ± 5%, and a dark-light cycle of 12 : 12 hours). They had free access to water and food. All the procedures with laboratory animals were carried out under the supervision of the National Committee on Ethics with the code of IR.FUMS.AEC.1401.005. A week after the rats had been purchased, all of them started to run for 10 min at a speed of 10 m/min for 5 days to get familiar with the treadmill. Then, we randomly assigned the animals into four groups: MI (*N* = 6), sham (*N* = 6), HIIT (*N* = 6), and HIIT+MI (*N* = 6). The MI and sham groups did not undergo the HIIT exercise program, but they were placed on the treadmill which did not move, so that the same conditions were provided for all rats. After the training period and MI induction, we anesthetized the rats with ketamine (100 mg/kg) and xylazine (10 mg/kg) injections.

Then, their heart blood samples were taken, collected in EDTA tubes, and centrifuged (SIGMA 2-15PK, 3100 rpm, 15°C, and 15 min) to measure the blood biomarkers. Then, their hearts were removed under sterile conditions and washed with PBS. A part of the heart tissue was placed in a formalin solution for staining and another part in an RNA solution for gene expression. The tissue and blood samples were taken to the laboratory for further experiments.

### 2.2. Measurement of V Peak (Peak Treadmill Running Speed)

The v-peak test was used to evaluate endurance capacity in this study [29]. The rats were first warmed up at a speed of 10 meters per minute for 5 minutes to experiment. Then, the test was started at a speed of 15 meters/minute for two minutes; we then increased the speed to 1.8 to 2 meters per minute every two minutes until the rats got tired (twice hitting the end of the treadmill electric shock or standing on it was considered exhaustion) [29]. This test was performed twice, at the beginning and end of the training period.

### 2.3. Physical Training Protocol

The v-peak test (maximum exercise performance test) was performed twenty-four hours after the last session of their familiarization with the treadmill. Then, after 48 hours, the rats started a 4-week training program which was done 5 sessions per week. A HIIT session consisted of a 5-minute warm-up (10-15 m/min of running) and 10 intervals of 1-minute running with a 2-minute rest between each interval (intensity of intervals: first week: 85% v peak, second week: 90% v peak, third week: 95% v peak, and fourth week: 100% v peak), and a 5 minutes cool-down (10 m/min of running). The v-peak test was repeated 24 hours after the last session of the training period [30, 31].

### 2.4. Induction of Myocardial Infarction

Forty-eight hours after the v-peak test at the end of the training period, isoproterenol (ISP), a beta-1 and beta-2 adrenergic receptor agonist, which was prepared in normal saline solution, was injected into the MI and the HIIT+MI groups (150 mg/kg), subcutaneously, on two consecutive days (24 h intervals) to induce myocardial infarction [32, 33]. To control the injection effect, we injected a placebo (distilled water) into the sham group. The rats were killed 48 hours after the second day of infarction induction.

### 2.5. Confirmation of Infarction

**Figure 1:**
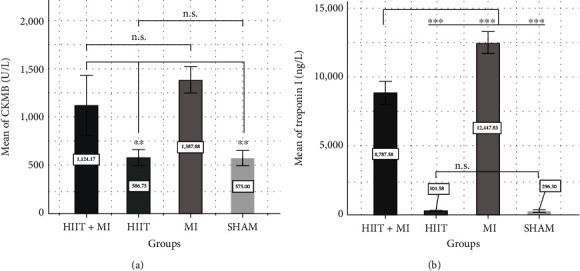
Cell damage markers in the experimental groups. (a) blood level of creatine kinase-MB (CK-MB), (b) blood level of troponin-I. ^∗∗^*P* < 0.01, ^∗∗∗^*P* < 0.001, n.s: not significant. (a, b) Exercise training attenuates MI-induced necrosis and fibrous.

To confirm the cardiac tissue damage, we measured blood levels of creatine kinase-MB (CK-MB) and troponin I (markers indicating cardiomyocyte necrosis) [32, 34], using the standard enzymatic method of the German Societies for Clinical Chemistry (DGKC) (Delta Darman Part, Iran), (the Vidas Troponin I test is a fast, precise, and sensitive method for the determination of cardiac troponin I), (Delta Darman Part, Iran); the activities of the enzymes were expressed in units of U/l and ng/l, respectively. All steps were performed according to the manufacturer's instructions. Hematoxylin-eosin staining (H&E) was used to evaluate the destruction of the heart tissue [32, 33, 35]. The samples were investigated using a light microscope with a magnification of ×40 after staining (Figure 1).

### 2.6. RT-PCR

To measure the gene expression of Mfn2 and Drp1 genes, we used a real-time polymerase chain reaction (RT-PCR method), which includes the following steps: (1) design and synthesis of primers were based on DNA sequence from NCBI (The National Center for Biotechnology Information) database using the Oligo Analyzer 1.0.2 tool, having minimum %GC, PCR product less than 220 bp, suitable BLAST results, and specificity above 85% (Takapouzist Co, Iran); (2) ribonucleic acid (RNA) was prepared by homogenizing heart tissue and extracting it with the total RNA extraction kit (Qiagen, German, Cat no: 74004); 3). The absorption ratio of 260/280 nm was used to determine the RNA quality of the samples in a NanoDrop 2000c (r) spectrophotometer (Thermo Scientific, Wilmington, DE, EUA); (4) complementary DNA synthesis (Thermo Fisher Scientific, USA, Cat no: k-1622) according to the manufacturer's instructions; and (5) amplification and detection by RT-PCR machine (Corbett, Qiagen, and German) by Rotor-Gene 6000. SYBR® Green RT-PCR Master Mix (Parstous, Iran) by a thermal program as follows: one cycle at 94°C for 10 minutes, 40 cycles at 95°C for 15 seconds, 55°C for 30 seconds, 72°C for 45 seconds, and 72°C for 5 minutes. Glyceraldehyde-3-phosphate dehydrogenase was used as a housekeeping gene for mRNA analysis. The logarithmic fold change (Log FC) was used to normalize the fold changes and analyze the gene expression data. GAPDH was used as a housekeeping gene for mRNA analysis. The primer sequences are shown in Table 1. It is worth noting that in the RT-PCR session the experimenters were blind.

### 2.7. Statistical Analysis

The log FC method was used to analyze the data in the present study. The Shapiro-Wilk test was used to determine the normal distribution of data, and one-way ANOVA and Bonferroni tests were used for data analysis. Statistical analysis was done using SPSS software version 20. All values were presented as means ± SD and *P* < 0.05. They were considered as statistically significant.

## 3. Results

### 3.1. Body Weight and V Peak

There was no significant difference in the weight and v peak (peak of speed) between the groups at the beginning of the training period (*P* > 0.05) (Table 2).

The results of this study, as shown in Figure 1, revealed a significant growth in creatine kinase-MB (CK-MB) and troponin-I after 4 weeks of HIIT and infarction (*P* < 0.001). The post hoc Bonferroni test showed that infract induction significantly increased these factors in the MI group and HIIT+MI group compared to the sham and HIIT groups (*P* < 0.05). Also, the results showed that exercise did not increase these cell damage markers in the HIIT+MI group compared to the MI group, which indicates the cardioprotective effect of the exercise.

### 3.2. Gene Expression

One-way analysis of variance test results showed that there was a significant difference between the expression levels of PGC-1*α* in cardiac tissue in different groups (*P* < 0.001). Also, the results of the Bonferroni post hoc test showed that there was a significant difference in the level of PGC-1*α* gene expression in the cardiac tissue between the sham group and the HIIT+MI, HIIT, and MI groups (*P* < 0.001). However, there was no significant difference between the HIIT and MI groups (*P* = 0.313), as well as the HIIT and HIIT+MI groups (*P* = 0.320). Moreover, based on the results of the one-way analysis of variance test, there was a significant difference between the expression levels of the TFAM gene in the cardiac tissue in different groups (*P* < 0.001).

The Bonferroni post hoc test showed that there was a significant difference in the expression level of the TFAM gene in the cardiac tissue between the sham group and the HIIT+MI, HIIT, and MI groups (*P* < 0.001). However, no significant difference was observed between the HIIT and HIIT+MI groups (*P* = 0.854). The results of the one-way analysis of variance test showed that there was a significant difference between the expression levels of the VEGF gene in the cardiac tissue in different groups (*p* < 0.001). The Bonferroni post hoc test revealed that there was a significant difference in the expression level of the VEGF gene in the heart tissue between the sham group and the HIIT+MI, HIIT, and MI groups (*P* < 0.001), while no significant difference was observed between the HIIT and MI groups (*P* = 0.078). Also, the results of the one-way analysis of variance test showed that there was no significant difference between the expression gene of miR-126 in the cardiac tissue in different groups (*P* = 0.127).

**Figure 2:**
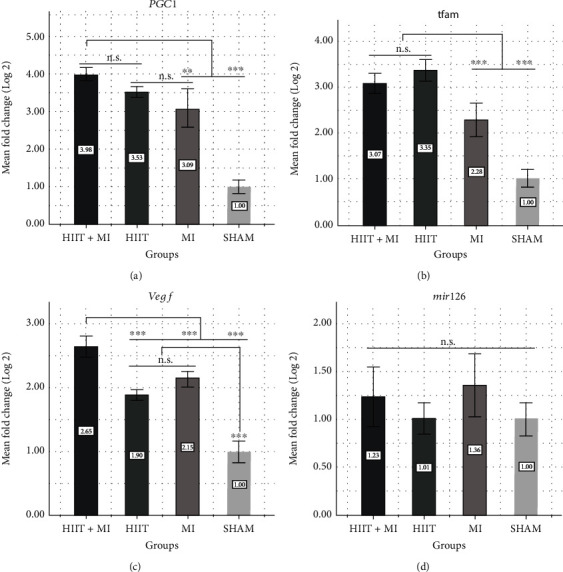
Gene expression of (a) peroxisome proliferator-activated receptor-gamma coactivating factor (PGC-1*α*), (b) mitochondrial transcription factor A (TFAM), (c) vascular endothelial growth factor (VEGF), and (d) microRNA 126 (miR-126) in experimental groups. ^∗∗^*P* < 0.01, ^∗∗∗^*P* < 0.001, n.s: not significant.

The Bonferroni post hoc test showed that there was no significant difference in the expression gene of miR-126 in the cardiac tissue between different groups of sham and HIIT+MI (*P* = 1), sham and HIIT (*P* = 1), sham and MI (*P* = 0.302), as well as MI and HIIT groups (*P* = 0.347) (Figure 2).

### 3.3. Exercise Training Reduces Cardiac Tissue Injury following MI

**Figure 3:**
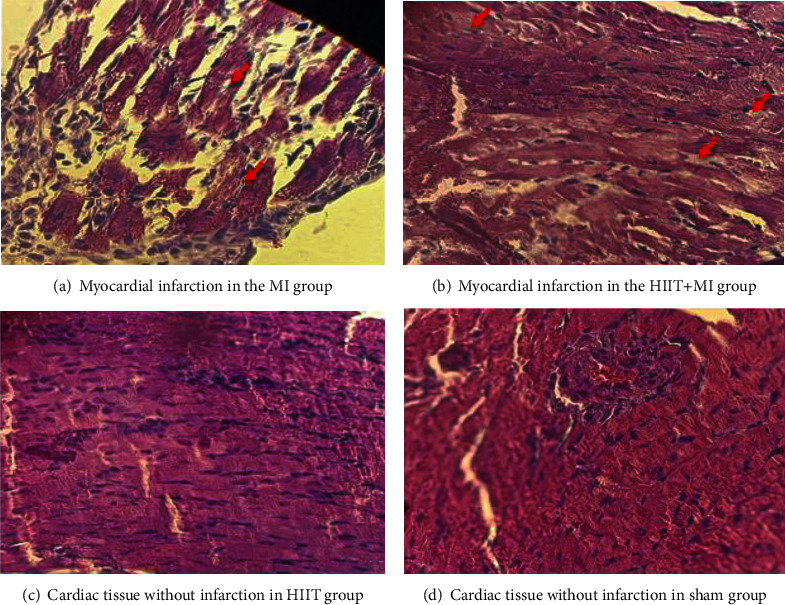
Necrosis in the heart tissue. (Scale bar = 40 *μ*m, 40x magnification).

As shown by hematoxylin and eosin (H&E) staining, exercise was associated with a decrease in cardiac tissue injury (Figure 3).

In the studied samples, the parts that took on less color and the nuclei of the cells lost their uniform appearance were designated as myocardial infarction areas. The red arrows indicate the myocardial infarction areas.

## 4. Discussion

Myocardial infarction is connected with rapid biochemical and metabolic changes in the heart tissue and leads to cardiac damage. [7]. Meanwhile, exercise training increases the protective ability of various tissues against possible injuries such as heart attacks. Tao et al. [36] showed that swimming training for 3 weeks reduced the infarct size as well as apoptosis and autophagy of cardiomyocytes. The cardioprotective effect of exercise is due to the improvement of glucose and lipid metabolism in the myocardium, as well as the adaptive increase of mitochondrial biogenesis, which is associated with the activation of PGC-1*α* [36]. The present study, in line with other studies [32, 37], showed that the rats that performed a short period of 4 weeks of HIIT training protocol were more resistant to MI.

As shown in Figure 3, the pathological results of the present study showed that isoproterenol injection caused severe heart tissue damage, which was very severe in the MI group, but moderate damage was observed in the HIIT+MI group. These severe pathological changes can include edema, accumulation of neutrophils, separation of fibers, bleeding, and collagen formation in the cardiac tissue. These findings are similar to the results of Farvin et al. and Tofighi et al. [38, 39]. You can also see in Figure 1 that CK-MB and troponin-I levels in the MI group increased more than the other three groups, which indicates a higher degree of damage in this group compared to the trained and sham groups. Actually, exercise and myocardial infarction are two factors that increase cardiac injury indicators such as CK MB and troponin I [40, 41], but it seems that none of the continuous or interval activities causes permanent heart damage, and the increase in heart damage indicators is the result of the unstable disorder of the heart cell membrane, and performing activities, especially continuous activities, will reduce this situation. It should be noted that, based on previous research, HIIT exercises can also produce the effects of continuous exercises. [42]. In this research, in both HIIT+MI and MI groups, after the induction of infarction, the level of these two indicators (CK-MB and troponin I) increased (Figures 1(a) and 1(b)), but in the HIIT+MI group, this increase is less, which is due to the protective effects of exercise. However, this decrease is not significant. Figure 3 shows less cardiac tissue damage in the HIIT+MI group after induction of myocardial infarction, which confirms the role of HIIT exercise in the protection of cardiac tissue. This result was consistent with those of Filho et al. and Nounou et al. [43, 44]. The results obtained in the present study showed the role of HIIT exercises in protecting the heart tissue and reducing cardiac tissue injury in the HIIT+MI group compared to the MI group (Figure 3).

In this study, it was also indicated that 4 weeks of HIIT training led to a significant increase in the expression of PGC-1*α* and TFAM in the HIIT and HIIT+MI groups compared to the sham group (*P* = 0.001). PGC-1*α* is a key regulator of mitochondrial biogenesis [42, 45, 46] that can be induced by exercise, and it plays an important role in myocardial metabolic control in heart diseases [47, 48]. Mitochondrial dysfunction is being observed in human cardiomyopathy and most animal models with heart failure. Mutations in mitochondrial DNA have been shown in humans after treatment with cardiotoxic therapies and in rodents after myocardial infarction. Therefore, these observations leave little doubt that defects in mitochondrial function can lead to heart disease. PGC-1*α* expression is suppressed in several models of heart failure [48]. On the other hand, the increase of PGC-1*α* stimulates the transcription of nuclear respiratory factor and leads to an increase in the expression of mitochondrial transcription factor A (TFAM) and other mitochondrial subunits of the electron transport chain [49]. TFAM is the target gene of NRF1, which plays an important role in the coordination of mutual reactions between the mitochondria and the nucleus [16]. This gene is a mitochondrial transcription factor that is a key activator in mitochondrial transcription [16]. When, during physical activity, the muscle contracts by receiving nerve impulses from motor neurons, calcium is released from the sarcoplasmic reticulum. Calcium acts as a strong secondary messenger and often works together with the calcium-binding protein known as calmodulin. In case where calcium acts as a secondary messenger, it stimulates various calcium-dependent enzymes such as calcium calmodulin kinase (camK) [50]. Then, calcium-dependent enzymes, including p38MAPK, specifically activate the transcription factor complexes [51]. In general, increasing the expression of the camK and p38MAPK genes increases the expression of PGC-1*α* and increases the expression of NRF-1, PGC-1*α*, and NRF-2, which in its turn increases the expression of TFAM. TFAM enters the mitochondrial nucleus and causes the regulation of mitochondrial DNA and the coded mitochondrial genes in the nucleus and ultimately causes mitochondrial biogenesis [52]. Targeted disruption of TFAM, especially in the cardiac muscle, leads to a significant decrease in electron transport capacity, spontaneous cardiomyopathy, and heart failure [53, 54]. Conversely, the increased expression of TFAM in the heart tissue protects against heart failure induced by myocardial infarction [55]. The observations emphasize the importance of maintaining mitochondrial integrity and the copy number as protection against heart disease.

Regarding the nonsignificant increase of PGC-1*α* in the HIIT+MI group compared to the HIIT group in the present study, it can be concluded that PGC-1*α* may be suppressed at first in some conditions such as heart attack, but it can finally act as a compensatory response to the rapid decrease in energy. The PGC-1*α* expression can be increased by ischemia [18, 56] and a waste of ATP [57, 58]. The reduction in myocardial tissue injury in the HIIT+MI group compared to the MI group is important here, which can be attributed to the role of exercise prepreparation in increasing the expression of PGC-1*α* and its downstream signaling pathways that lead to mitochondrial biogenesis and angiogenesis (Figures 3(a) and 3(b)).

Also, angiogenesis is one of the changes that occur in the vascular structure to relieve stress conditions during exercise, including high-intensity interval training (HIIT), and VEGF is known as the strongest mitogen for the endothelial cells among the angiogenic factors [22]. It has been found that PGC-1*α* is also involved in angiogenesis resulting from exercise; thus, the increase in angiogenesis in the skeletal muscle does not occur in animal samples of transgenic rats lacking PGC-1*α* [19]. In other words, beta-adrenergic stimulations lead to the launch of different programs of angiogenic factors such as VEGF that require PGC-1*α* itself. The ERR*α*, PGC-1*α*, and VEGF pathways play a role in muscle angiogenesis, in the first step by increasing the expression of PGC-1*α* [19]. In research, Ding et al. found that after 3 weeks of exercise, VEGF mRNA levels increased significantly, which led to angiogenesis and stroke reduction [59]. In the present study, it is also shown that HIIT exercises significantly increase the expression of VEGF in the HIIT and HIIT+MI groups compared to the sham group (*P* = 0.001), and these results are consistent with those of Bayati et al. [60], Taylor et al. [61], and Yazdanian et al. [62].

Recent studies have shown the important role of microRNAs in the response of the cardiovascular system to injury, inflammation, and stress [63]. One of these special microRNAs is miR-126, which is mostly expressed in the cardiac muscle. Also, miR-126 is known as an endothelial-specific miRNA because it plays the most important and best role in controlling angiogenesis and vascular integrity [27]. miR-126 directly suppresses two negative regulators of the VEGF pathway. These two pathways are Sprouty-related protein 1 (Spred-1) which is an intracellular suppressor of the Ras/MAPK pathway and phosphoinositol-3 kinase regulatory subunit 2 (PIK3R2) which regulates the activity of the PIK3/Akt/eNOS pathway negatively [20]. In a study, Fish et al. also found that the phosphorylation of ERK 1/2 and AKT caused by VEGF was weakened in the cells in which miR-126 was disrupted [64]. Da Silva Jr. et al. [27] studied the role of aerobic swimming exercise on miR-126 expression. This study showed that aerobic exercise increased the expression of miR-126, and this can be related to cardiac angiogenesis caused by exercise, directly regulating the VEGF pathway and indirectly regulating its targets such as MAPK, PI3K/Akt/eNOS [27]. In line with the various research mentioned in the present study, HIIT exercises for 4 weeks increased miR-126 in the cardiac muscle tissue of rats in the HIIT and HIIT+MI groups compared to the sham group; however, this increase was not significant. It can be attributed to the time of measurement or the duration of the exercises. Four weeks of HIIT training reduced cardiac tissue injury before inducing MI (Figure 3). Damage reduction is associated with angiogenesis that is possibly induced by post-exercise angiogenic factors.

In general, the results of the present study showed that four weeks of HIIT training led to an increase in the expression of VEGF, TFAM, PGC-1*α*, and mir-126 genes in the heart tissue of male Wistar rats, which probably reduces the cardiac tissue injury by increasing mitochondrial biogenesis and angiogenesis following isoproterenol injection. The scope of the findings of this study is limited to rats, and more relevant clinical research is needed before HIIT can be recommended as a heart protection program.

## 5. Conclusions

According to the results obtained in this study, it can be said that HIIT exercises exert cardioprotective effects to reduce cardiac tissue injury and decrease the apoptosis of the heart in MI; these effects caused an increase in mitochondrial biogenesis and angiogenesis by inducing increased expression of VEGF, TFAM, PGC-1*α*, and miR-126 genes in the cardiac tissue. It is necessary to mention that the protective effect of exercise against acute myocardial injury following MI should be more complex than myocardial energy metabolism and mitochondrial biogenesis, and angiogenesis.

Furthermore, although the current study showed that exercise protected against acute myocardial injury after MI, the effect of exercise on improving adverse remodeling after permanent coronary artery occlusion remains to be investigated. Also, the study was conducted on male rats only, and the findings may not be generalizable to humans. Therefore, more clinical research in this field is suggested.

## Figures and Tables

**Table 1:** RT-PCR primer sequences.

PGC-1*α*	Forward: 5′-CTAGAGGATGGCTGCACTAAACAC-3′
	Reserve: 5′-AAGCAAACAGGGCCAATGTC-3′
TFAM	Forward: 5′-GAAGGGAATGGGAAAGGTAGA-3′
	Reverse: 5′-AACAGGACATGGAAAGCAGAT-3′
VEGF	Forward: 5′ -CTTTCTGCTCTCTTGGGTGC-3′
	Reverse: 5′-GTAGACGTCCATGAACTTCAC-3′
miR-126	Forward: 5′ -GCGTCGTACCGTGAGTAAT-3′
	Reverse: 5′-GTCGTATCCAGTGCGTGTCGTGGAGTCGGCAATTGCACTGGATACGACCGCATT-3′

**Table 2:** Body weight and v peak of the rats in groups.

	HIIT+MI	HIIT	MI	SHAM
Weight (pre) (gr)	276.83 ± 20.80	256 ± 32.02	274.75 ± 27.64	270.25 ± 1.84
Weight (post) (gr)	324 ± 21.56	303.50 ± 34.24	311.87 ± 31.95	315 ± 27.99
V peak (pre) (M/min)	41.32 ± 3.50	41.98 ± 3.41	40.86 ± 2.74	40.67 ± 1.61
V peak (post) (M/min)	50.90 ± 3.52	53.65 ± 3.06	42.55 ± 2.05	42.55 ± 2.15

## Data Availability

The data used to support the findings of this study are included within the article.

## References

[1] Nordlie M. A., Wold L. E., Kloner R. A. (2005). Genetic contributors toward increased risk for ischemic heart disease. *Journal of Molecular and Cellular Cardiology*.

[2] Arany Z., Novikov M., Chin S., Ma Y., Rosenzweig A., Spiegelman B. M. (2006). Transverse aortic constriction leads to accelerated heart failure in mice lacking PPAR-*γ* coactivator 1*α*. *Proceedings of the National Academy of Sciences of the United States of America*.

[3] Huss J. M., Kelly D. P. (2004). Nuclear receptor signaling and cardiac energetics. *Circulation Research*.

[4] Honda T., Kaikita K., Tsujita K. (2008). Pioglitazone, a peroxisome proliferator-activated receptor-*γ* agonist, attenuates myocardial ischemia-reperfusion injury in mice with metabolic disorders. *Journal of Molecular and Cellular Cardiology*.

[5] Shiomi T., Tsutsui H., Hayashidani S. (2002). Pioglitazone, a peroxisome proliferator–activated receptor-*γ* agonist, attenuates left ventricular remodeling and failure after experimental myocardial infarction. *Circulation*.

[6] Dominy J. E., Puigserver P. (2013). Mitochondrial biogenesis through activation of nuclear signaling proteins. *Cold Spring Harbor Perspectives in Biology*.

[7] Borges J. P., Lessa M. A. (2015). Mechanisms involved in exercise-induced cardioprotection: a systematic review. *Arquivos Brasileiros de Cardiologia*.

[8] Bayati M., Gharakhanloo R., Farzad B. (2015). Adaptations of physiological performance following high-intensity interval training. *Sport Physiology*.

[9] Bayati M., Farzad B., Gharakhanlou R., Agha-Alinejad H. (2011). A practical model of low-volume high-intensity interval training induces performance and metabolic adaptations that resemble 'all-out' sprint interval training. *Journal of Sports Science & Medicine*.

[10] Hood M. S., Little J. P., Tarnopolsky M. A., Myslik F., Gibala M. J. (2011). Low-volume interval training improves muscle oxidative capacity in sedentary adults. *Medicine and Science in Sports and Exercise*.

[11] Little J. P., Safdar A., Wilkin G. P., Tarnopolsky M. A., Gibala M. J. (2010). A practical model of low-volume high-intensity interval training induces mitochondrial biogenesis in human skeletal muscle: potential mechanisms. *The Journal of Physiology*.

[12] Burgomaster K. A., Howarth K. R., Phillips S. M. (2008). Similar metabolic adaptations during exercise after low volume sprint interval and traditional endurance training in humans. *The Journal of Physiology*.

[13] Yin W., Signore A. P., Iwai M., Cao G., Gao Y., Chen J. (2008). Rapidly increased neuronal mitochondrial biogenesis after hypoxic-ischemic brain injury. *Stroke*.

[14] Kordi M. R., Nekouei A., Shafiee A., Hadidi V. (2015). The effect of eight weeks high intensity aerobic continuous and interval training on gene expression of vascular endothelial growth factor in soleus muscle of healthy male rats. *Arak Medical University Journal*.

[15] Taheri R., Mirzaei B., Demirchi A. (2021). The effect of 8 weeks of interval and resistance training on expression PGC 1*α*, AMPK, TFAM elderly rat heart cells. *Medical Journal of Mashhad University of Medical Sciences*.

[16] Wang C., Li Z., Lu Y. (2006). Cyclin D1 repression of nuclear respiratory factor 1 integrates nuclear DNA synthesis and mitochondrial function. *Proceedings of the National Academy of Sciences of the United States of America*.

[17] Alavi S. Y., Mirdar S. (2021). The comparison of eight weeks of HIIT and BFR on mitochondrial biogenesis and angiogenesis markers in vastus lateralis muscle of amateur male runners. *The Scientific Journal of Rehabilitation Medicine*.

[18] Arany Z., Foo S.-Y., Ma Y. (2008). HIF-independent regulation of VEGF and angiogenesis by the transcriptional coactivator PGC-1*α*. *Nature*.

[19] Chinsomboon J., Ruas J., Gupta R. K. (2009). The transcriptional coactivator PGC-1*α* mediates exercise-induced angiogenesis in skeletal muscle. *Proceedings of the National Academy of Sciences of the United States of America*.

[20] Leosco D., Rengo G., Iaccarino G. (2008). Exercise promotes angiogenesis and improves *β*-adrenergic receptor signalling in the post-ischaemic failing rat heart. *Cardiovascular Research*.

[21] Mounier R., Pialoux V., Roels B. (2009). Effect of intermittent hypoxic training on HIF gene expression in human skeletal muscle and leukocytes. *European Journal of Applied Physiology*.

[22] Prior B. M., Yang H., Terjung R. L. (2004). What makes vessels grow with exercise training?. *Journal of Applied Physiology*.

[23] Adair T. H., Cotten R., Gu J.-W. (2005). Adenosine infusion increases plasma levels of VEGF in humans. *BMC Physiology*.

[24] Djonov V., Baum O., Burri P. H. (2003). Vascular remodeling by intussusceptive angiogenesis. *Cell and Tissue Research*.

[25] Gavin T. P., Stallings H. W., Zwetsloot K. A. (2005). Lower capillary density but no difference in VEGF expression in obese vs. lean young skeletal muscle in humans. *Journal of Applied Physiology*.

[26] Latronico M. V., Condorelli G. (2011). Therapeutic use of microRNAs in myocardial diseases. *Current Heart Failure Reports*.

[27] Da Silva N. D., Fernandes T., Soci U. P., Monteiro A. W., Phillips M. I., De Oliveira E. M. (2012). Swimming training in rats increases cardiac microRNA-126 expression and angiogenesis. *Medicine and Science in Sports and Exercise*.

[28] Verboven M., Cuypers A., Deluyker D. (2019). High intensity training improves cardiac function in healthy rats. *Scientific Reports*.

[29] Høydal M. A., Wisløff U., Kemi O. J., Ellingsen Ø. (2007). Running speed and maximal oxygen uptake in rats and mice: practical implications for exercise training. *European Journal of Preventive Cardiology*.

[30] Ahmadi A., Kashef M., Rajabi H., Salehpour M. (2023). Effects of exercise preconditioning on NLRP3 and mitochondrial fission in isoproterenol-induced myocardial infarcted rats. *Comparative Clinical Pathology*.

[31] Hoshino D., Yoshida Y., Kitaoka Y., Hatta H., Bonen A. (2013). High-intensity interval training increases intrinsic rates of mitochondrial fatty acid oxidation in rat red and white skeletal muscle. *Applied Physiology, Nutrition, and Metabolism*.

[32] Ghanimati R., Rajabi H., Ramezani F., Ramez M., Bapiran M., Nasirinezhad F. (2020). The effect of preconditioning with high-intensity training on tissue levels of G-CSF, its receptor and C-kit after an acute myocardial infarction in male rats. *BMC Cardiovascular Disorders*.

[33] Shukla S. K., Sharma S. B., Singh U. R. (2015). *β*-Adrenoreceptor agonist isoproterenol alters oxidative status, inflammatory signaling, injury markers and apoptotic cell death in myocardium of rats. *Indian Journal of Clinical Biochemistry*.

[34] Chowdhury M. A., Sholl H. K., Sharrett M. S. (2019). Exercise and cardioprotection: a natural defense against lethal myocardial ischemia–reperfusion injury and potential guide to cardiovascular prophylaxis. *Journal of Cardiovascular Pharmacology and Therapeutics*.

[35] El-Ashmawy N. E., Khedr N. F., Shaban M. N., Al-Ashmawy G. M. (2022). Diallyl trisulfide modulated autophagy in isoproterenol induced acute myocardial infarction. *Clinical Phytoscience*.

[36] Tao L., Bei Y., Lin S. (2015). Exercise training protects against acute myocardial infarction via improving myocardial energy metabolism and mitochondrial biogenesis. *Cellular Physiology and Biochemistry*.

[37] Rahimi M., Shekarforoush S., Asgari A. R. (2015). The effect of high intensity interval training on cardioprotection against ischemia-reperfusion injury in wistar rats. *EXCLI Journal*.

[38] Farvin K., Surendraraj A., Anandan R. (2010). Protective effect of squalene on certain lysosomal hydrolases and free amino acids in isoprenaline-induced myocardial infarction in rats. *International Journal of Pharmacology*.

[39] Tofighi A., Ebrahimi Kalan A., Jamali Qarakhanlou B. (2017). The effect of resveratrol supplementation and aerobic training on cardiac tissue alteration of rats with acute myocardial infarction. Iranian. *Journal of Physiology and Pharmacology*.

[40] Nalcakan G. R. (2014). The effects of sprint interval vs. continuous endurance training on physiological and metabolic adaptations in young healthy adults. *Journal of Human Kinetics*.

[41] Kordi M. R., Khodayari B., Gaeini A., Reza N. (2018). The comparison of three exercise protocols on specific biochemical markers of cardiac cells in overweight men. *Journal of Applied Exercise Physiology*.

[42] Heiat F., Ghanbarzadeh M., Shojaeifard M., Ranjbar R. (2021). Influence de l'Interval training de haute intensite (HIIT) sur les niveaux d'expression des proteines PGC-1*α* et SIRT3 et l'indice de vieillissement (GSH/ GSSG) dans les muscles lent (SOL) et rapide (EDL) de rats. *Science & Sports*.

[43] Filho H. G. L., Ferreira N. L., de Sousa R. B., de Carvalho E. R., Lobo P. L. D., Filho J. G. L. (2011). Experimental model of myocardial infarction induced by isoproterenol in rats. *Brazilian Journal of Cardiovascular Surgery*.

[44] Nounou H. A., Deif M. M., Shalaby M. A. (2012). Effect of flaxseed supplementation and exercise training on lipid profile, oxidative stress and inflammation in rats with myocardial ischemia. *Lipids in Health and Disease*.

[45] Heiat F., Heiat M., Shojaeifard M. (2021). Changes in mitochondrial biogenesis and fatty liver indicators in rat following continuous and high intensity interval training. *The Journal of Sports Medicine and Physical Fitness*.

[46] Heiat F., Ghanbarzadeh M., Ranjbar R., Shojaeifard M. (2020). Continuous swimming training arises a remarkable effect on some longevity biomarkers in rat skeletal muscles. *Annals of Applied Sport Science*.

[47] Ventura-Clapier R., Garnier A., Veksler V. (2008). Transcriptional control of mitochondrial biogenesis: the central role of PGC-1. *Cardiovascular Research*.

[48] Rowe G. C., Jiang A., Arany Z. (2010). PGC-1 coactivators in cardiac development and disease. *Circulation Research*.

[49] Irrcher I., Adhihetty P. J., Sheehan T., Joseph A.-M., Hood D. A. (2003). PPAR*γ* coactivator-1*α* expression during thyroid hormone-and contractile activity-induced mitochondrial adaptations. *American Journal of Physiology-Cell Physiology*.

[50] Hardie D. G. (2007). AMP-activated/SNF1 protein kinases: conserved guardians of cellular energy. *Nature Reviews Molecular Cell Biology*.

[51] Meirhaeghe A., Crowley V., Lenaghan C. (2003). Characterization of the human, mouse and rat PGC1beta (peroxisome-proliferator-activated receptor-gamma co-activator 1beta) gene in vitro and in vivo. *Biochemical Journal*.

[52] Brown G. C., Murphy M. P., Jornayvaz F. R., Shulman G. I. (2010). Regulation of mitochondrial biogenesis. *Essays in Biochemistry*.

[53] Li H., Wang J., Wilhelmsson H. (2000). Genetic modification of survival in tissue-specific knockout mice with mitochondrial cardiomyopathy. *Proceedings of the National Academy of Sciences of the United States of America*.

[54] Wang J., Wilhelmsson H., Graff C. (1999). Dilated cardiomyopathy and atrioventricular conduction blocks induced by heart-specific inactivation of mitochondrial DNA gene expression. *Nature Genetics*.

[55] Ikeuchi M., Matsusaka H., Kang D. (2005). Overexpression of mitochondrial transcription factor a ameliorates mitochondrial deficiencies and cardiac failure after myocardial infarction. *Circulation*.

[56] Chawla A. (2010). Control of macrophage activation and function by PPARs. *Circulation Research*.

[57] Rohas L. M., St-Pierre J., Uldry M., Jäger S., Handschin C., Spiegelman B. M. (2007). A fundamental system of cellular energy homeostasis regulated by PGC-1*α*. *Proceedings of the National Academy of Sciences of the United States of America*.

[58] Jäger S., Handschin C., St-Pierre J., Spiegelman B. M. (2007). AMP-activated protein kinase (AMPK) action in skeletal muscle via direct phosphorylation of PGC-1*α*. *Proceedings of the National Academy of Sciences of the United States of America*.

[59] Ding Y.-H., Luan X.-D., Li J. (2004). Exercise-induced overexpression of angiogenic factors and reduction of ischemia/reperfusion injury in stroke. *Current Neurovascular Research*.

[60] Bayati M., Gharakhanlou R., Nikkhah M., Amani Shalamzari S. (2018). The effect of four weeks of high-intensity interval training on PGC-1*α* and VEGF protein contents in skeletal muscle of active men. *Journal of Arak University of Medical Sciences*.

[61] Taylor C. W., Ingham S. A., Hunt J. E., Martin N. R., Pringle J. S., Ferguson R. A. (2016). Exercise duration-matched interval and continuous sprint cycling induce similar increases in AMPK phosphorylation, PGC-1*α* and VEGF mRNA expression in trained individuals. *European Journal of Applied Physiology*.

[62] Yazdanian N., Asad M. R., Rahimi M. (2018). The effect of high intensity interval training and moderate-intensity continuous training on PGC1*α* and VEGF in heart muscle of male wistar rats. *Sport Physiology*.

[63] Latronico M. V., Condorelli G. (2009). MicroRNAs and cardiac pathology. *Nature Reviews Cardiology*.

[64] Fish J. E., Santoro M. M., Morton S. U. (2008). miR-126 regulates angiogenic signaling and vascular integrity. *Developmental Cell*.

